# Emerging molecular classifications and therapeutic implications for gastric cancer

**DOI:** 10.1186/s40880-016-0111-5

**Published:** 2016-05-27

**Authors:** Tao Chen, Xiao-Yue Xu, Ping-Hong Zhou

**Affiliations:** Endoscopy Center, Zhongshan Hospital of Fudan University, 180 Fenglin Rd, Shanghai, 200032 P. R. China; Endoscopy Research Institute, Fudan University, Shanghai, 200032 P. R. China

**Keywords:** Gastric cancer, Molecular classification, Personalized therapy, The Cancer Genome Atlas research network

## Abstract

Gastric cancer (GC) is a highly aggressive and life-threatening malignancy. Even with radical surgical removal and front-line chemotherapy, more than half of GCs locally relapse and metastasize at a distant site. The dismal outcomes reflect the ineffectiveness of a one-size-fits-all approach for a highly heterogeneous disease with diverse etiological causes and complex molecular underpinnings. The recent comprehensive genomic and molecular profiling has led to our deepened understanding of GC. The emerging molecular classification schemes based on the genetic, epigenetic, and molecular signatures are providing great promise for the development of more effective therapeutic strategies in a more personalized and precise manner. To this end, the Cancer Genome Atlas (TCGA) research network conducted a comprehensive molecular evaluation of primary GCs and proposed a new molecular classification dividing GCs into four subtypes: Epstein-Barr virus-associated tumors, microsatellite unstable tumors, genomically stable tumors, and tumors with chromosomal instability. This review primarily focuses on the TCGA molecular classification of GCs and discusses the implications on novel targeted therapy strategies. We believe that these fundamental findings will support the future application of targeted therapies and will guide our efforts to develop more efficacious drugs to treat human GCs.

## Background

Gastric cancer (GC) is the fourth most common cancer diagnosed worldwide [1] and the second leading cause of cancer-related death, accounting for approximately 10% of all cancer deaths [2, 3]. In China, GC is among the three most common cancers [4]. However, in Western countries, although distal GCs are now uncommon, the incidence of cancers located in the gastric cardia and gastroesophageal junction is steadily increasing [5]. Currently, surgery remains the only curative treatment strategy; however, more than half of radical GCs locally relapse or distantly metastasize [6]. This highly malignant behavior is mainly due to the complexity of GC progression, including inherited and environmental factors, such as habits, diet, virus infection [especially *Helicobacter pylori* and Epstein-Barr virus (EBV) infection], and genomics [7]. Therefore, GC is a more heterogeneous disease than previously thought. Using the traditional histological classifications that are well accepted, it is difficult to judge the genetic and epigenetic alterations for precise diagnosis and treatment and to predict prognosis and clinical outcomes.

The latest advances in molecular platforms, such as next-generation sequencing (NGS), have led to the development of comprehensive profiling of GCs. The results have deepened our understanding of GC biology and expanded the possibility of novel experimental treatments for GCs. In this article, we review the current knowledge regarding the emerging molecular classifications of GCs, primarily focusing on the new Cancer Genome Atlas (TCGA) research network classification of GCs, and discuss the therapeutic implications.

### Gastric cancer heterogeneity and molecular classifications

A large body of evidence supports the idea that heterogeneity in cancer not only exists between different patients (inter-tumor heterogeneity) but also occurs within a single patient (intra-tumor heterogeneity). In a study on genetic heterogeneity of GCs, erb-b2 receptor tyrosine kinase 2 (*ERBB2*, also known as *HER2*) was amplified in 19 (17.4%) of 109 samples [8]. Meanwhile, intra-tumor heterogeneity was also identified in 50%–80% of primary GCs, and the *HER2* amplification occurred in these GCs [8]. The results of this study underline the importance of obtaining multiple biopsies from different tumor areas for diagnostic purposes. This notion might be particularly important when a highly heterogeneous target gene is analyzed. Together, it calls for a new classification based on the molecular characteristics of GC given that GC has been recognized as a much more heterogeneous disease than previously thought [9, 10].

**Fig. 1:**
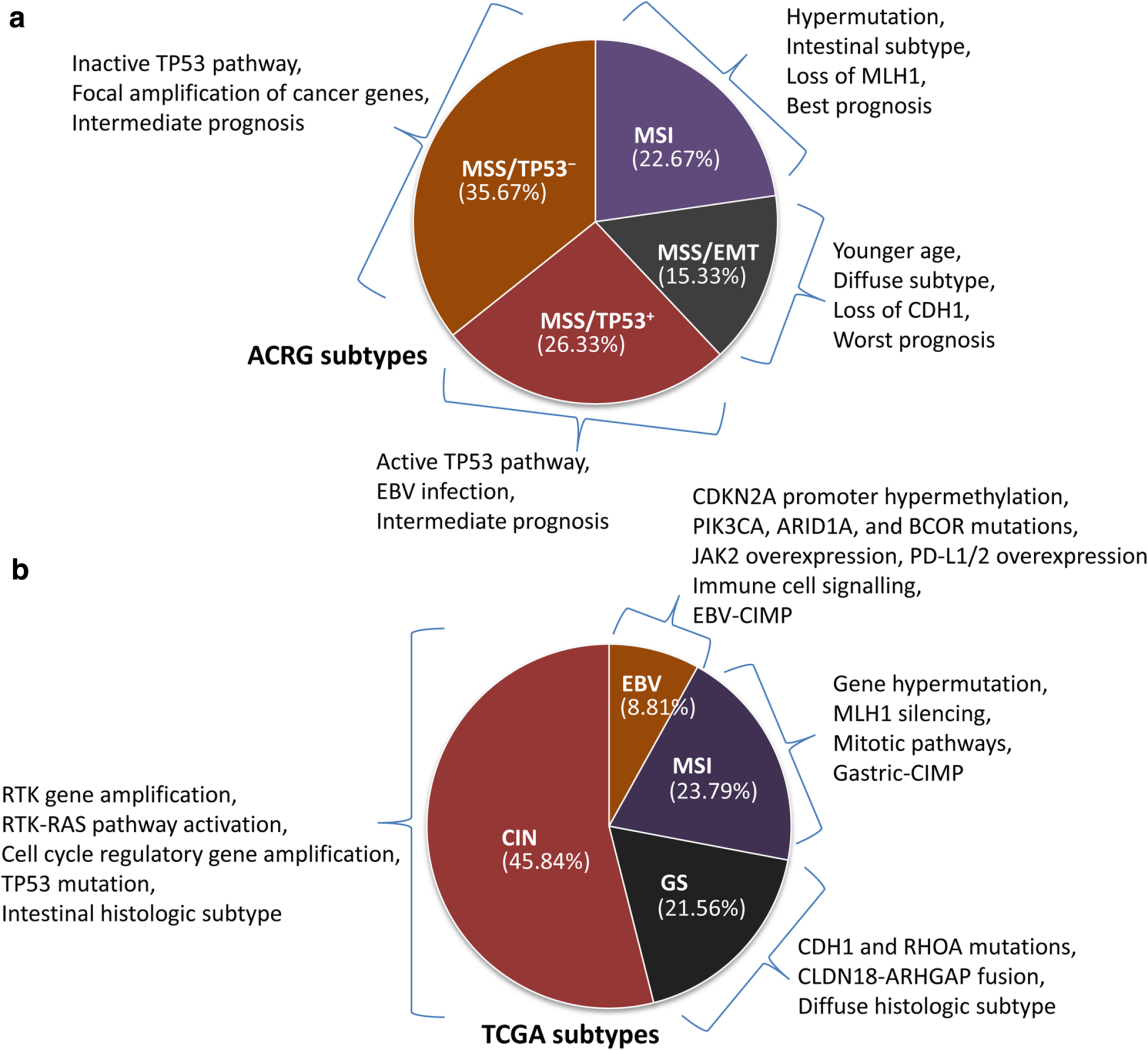
Molecular classifications of gastric cancers (GCs): **a** the Asian Cancer Research Group (ACRG) classification; **b** the Cancer Genome Atlas (TCGA) classification. *MSS* microsatellite stable, *TP53* tumor protein 53, *MSI* microsatellite instable, *EMT* epithelial-mesenchymal transition, *MLH1* mutL homolog 1, *CDH1* cadherin 1, *EBV* Epstein-Barr virus, *CIN,* chromosomal instability, *GS* genomically stable, *RTK* receptor tyrosine kinase, *RAS* resistance to audiogenic seizures, *CDKN2A* cyclin-dependent kinase inhibitor 2A, *PIK3CA* phosphatidylinositol-4,5-bisphosphate 3-kinase catalytic subunit alpha, *ARID1A* AT-rich interactive domain 1A, *BCOR* BCL6 corepressor, *JAK2* janus kinase 2, *PD-L* programmed cell death ligand, *CIMP* CpG island methylator phenotype, *RHOA* ras homolog family member A, *CLDN18* claudin 18, *ARHGAP* Rho GTPase-activating protein 6

Traditionally, GCs are commonly classified into intestinal and diffuse subtypes according to the Lauren classification or papillary, tubular, mucinous (colloid), and poorly cohesive carcinomas according to the World Health Organization (WHO) classification (Table 1). Based on the integrated genetic characteristics of GCs, the Asian Cancer Research Group (ACRG) analyzed gene expression in 300 primary gastric tumors and established four molecular subtypes (Fig. 1): the microsatellite stable (MSS)/epithelial-mesenchymal transition (EMT) subtype, microsatellite instable (MSI) subtype, MSS/tumor protein 53 (TP53)^+^ subtype, and MSS/TP53^−^ subtype [11–13]. Overall, MSS/EMT tumors have the highest frequency of recurrence (63%) with the worst prognosis; the MSI subtype has the lowest frequency of recurrence (22%) with the best overall prognosis; the MSS/TP53^+^ and MSS/TP53^−^ subtypes have intermediate prognosis and recurrence rates. Therefore, the ACRG provides a molecular classification that focuses on the association between genetic profiling and clinical outcomes. In 2014, TCGA performed a comprehensive molecular characterization of GCs from 295 patients who had not been treated with prior chemotherapy or radiotherapy [14, 15]. Concerning increasing evidence of GC heterogeneity, TCGA integrated the results of genetic alterations and proposed a molecular classification of GCs into four major subtypes: EBV-associated tumors, MSI tumors, genomically stable (GS) tumors, and tumors with chromosomal instability (CIN) (Fig. 1). In this article, we review and discuss the TCGA classification and its therapeutic implications.

**Table 1:** Pathologic classifications of gastric cancers (GCs)

Classification	Subtype	Characteristics
Lauren	Intestinal	Recognizable glands, arise on a background of intestinal metaplasia
	Diffuse	Poor cohesiveness, round small cells, diffuse infiltration in the gastric wall with little or no gland formation
WHO	Tubular adenocarcinoma	Prominent dilated or slit-like, branching tubules
	Papillary adenocarcinoma	Well-differentiated tumor cells, exophytic growth
	Mucinous adenocarcinoma	Extracellular mucinous pools
	Signet-ring cell carcinoma	Cells lie scattered in the lamina propria, wide distances between the pits and glands

*WHO* the World Health Organization

### The TCGA classification of gastric cancers

#### Epstein-Barr virus-associated gastric cancer (EBVaGC)

EBV was discovered 50 years ago from Burkitt’s lymphoma [16, 17] and is carried in the blood circulation without symptoms in 90% of the adult population [18]. However, for reasons yet to be further identified, EBV may affect epithelial cells and become carcinogenic. It is estimated that EBV is associated with 2% of all human tumors, including nasopharyngeal carcinoma [19], another major cancer type that is unique to the Chinese population especially in the southern areas, such as Guangdong province. In recent years, it has been increasingly recognized that the majority of GCs are associated with infectious agents, including EBV [20]. EBV is found within malignant epithelial cells in 9% of GCs [21]. Given that EBV was first found in GC cells in 1990, the relationship between EBV and GC has become a research hotspot [22]. It is reported that the major molecular characteristic of EBVaGCs is CpG island promoter methylation of GC-related genes [20]. The expression of EBV latent membrane protein 2A (*LMP2A*) may result in the promotion of DNA methylation through inducing signal transducer and activator of transcription 3 (STAT3) phosphorylation and subsequent transcription of DNA methyltransferase 1 (*DNMT1*) [23]. TCGA reported the special characteristic of EBVaGCs [14]. They found that most of these cancers were present in the gastric fundus or body. They also demonstrated that more DNA hypermethylations occur in EBVaGCs compared with other subtypes. EBV-associated DNA hypermethylations involve both promoter and non-promoter CpG islands. Cyclin-dependent kinase inhibitor 2A (*CDKN2A*) promoter hypermethylation was demonstrated in all EBVaGCs, whereas mutL homolog 1 (*MLH1*) hypermethylation was not noted.

In the aspect of somatic gene alterations, strong predilection for phosphatidylinositol-4,5-bisphosphate 3-kinase, catalytic subunit alpha (*PIK3CA*) mutation was observed in EBVaGCs, and approximately 5%–10% of all GCs exhibited a *PIK3CA* mutation. In addition to *PIK3CA* mutation, EBVaGCs had frequent AT-rich interactive domain 1A (*ARID1A*) mutation (55%) and BCL6 corepressor (*BCOR*) mutation (23%) and rarely had a *TP53* mutation. Chen et al. [24] identified *TP53* and *ARID1A* mutations as markers for high clonality and low clonality subtypes of GC in Chinese patients, respectively. Simultaneously, a novel recurrent amplification locus containing janus kinase 2 (*JAK2*), *CD274*, and programmed cell death 1 ligand 2 (*PDCD1LG2*) was identified in EBVaGCs. These genes encode JAK2, programmed cell death 1 ligand (PD-L1), and programmed cell death 2 ligand (PD-L2), separately. JAK2 is used by several class I cytokine receptors, including growth hormone (GH), erythropoietin (EPO), and prolactin. These receptors primarily use JAK2 to activate STAT to regulate gene transcription. PD-L1/2 and their receptors PD-1/2 are involved in immune checkpoints. Thus, the EBV subtype can be a good candidate for testing immunotherapy.

#### MSS and CIN gastric cancers

Knowledge on the molecular mechanisms indicates that two major genomic instability pathways, MSI and CIN, are involved in the pathogenesis of GCs. MSI is caused by widespread replication errors in simple repetitive microsatellite sequences due to the defects in mismatch repair genes. MSI has been recognized as an early change in GC carcinogenesis [25]. TCGA reported that the MSI subgroup represented 21% of GCs. In addition, MSI cases were characterized by accumulation of mutations in *PIK3CA*, *ERBB3*, *HER2*, and epidermal growth factor receptor (*EGFR*), but MSI cancers generally lacked targetable amplifications. Importantly, *B*-*Raf* (V600E) mutation was not identified in MSI GCs but was commonly found in colorectal cancer.

CIN involves the unequal distribution of DNA to daughter cells upon mitosis and results in the loss or gain of chromosome during cell division [26]. CIN is a more common pathway that may comprise clinicopathologically and molecularly heterogeneous cancers [27]. GC has been demonstrated to exhibit significant abnormalities in DNA content. Copy number gains at 8q, 12q, 13q, 17q, and 20q and copy number losses at 3p, 4q, 5q, 15q, 16q, and 17q are frequently noted in GCs [28–31]. In addition to chromosomal gains and losses, CIN contributes to focal gene amplifications as well. In TCGA data, the CIN subtype represented 50% of GCs and showed elevated frequency in the gastroesophageal junction/cardia. Genomic amplifications of genes that encode receptor tyrosine kinases (RTKs) were identified in the CIN subtype. A new finding is that elevated phosphorylation of EGFR (pY1068) was observed in the CIN subtype and consistent with amplification of *EGFR* [14]. In addition, amplifications of cell cycle genes Cyclin E1 (*CCNE1*), Cyclin D1 (*CCND1*), and Cyclin-dependent kinase 6 (*CDK6*) have been noted in CIN tumors. These gene amplifications could be the molecular basis of therapeutic monoclonal antibodies and targeted agents given that their amplifications can lead to excessive cancer cell growth.

#### GS gastric cancer

In the TCGA study, GS tumors represented 19.6% of GCs and were enriched with diffuse histological variant. Fifteen percent of ras homolog family member A (*RHOA*) mutations were enriched in this diffuse GC subtype. The role of RHOA in cell motility highlights the contribution of RHOA modification to altered cell adhesion in the carcinogenesis of diffuse GCs. In addition, mutations in cadherin 1 (*CDH1*) have also been detected in diffuse GCs. *CDH1* germline mutations underlie hereditary diffuse GCs and are associated with poorly differentiated GCs and poor prognosis. In the GS subtype, a recurrent interchromosomal translocation between claudin 18 (*CLDN18*) and Rho GTPase-activating protein 6 (*ARHGAP26*) was also identified. RNA sequencing data from the TCGA cohort identified *CLDN18*-*ARHGAP26* fusion in 3.05% of GCs as well as CLDN18 fusion to the homologous GTPase-activating protein encoded by *ARHGAP6* in 2 cases. This type of fusion gene may represent a class of gene fusions in cancers that establish pro-oncogenic tumor growth and prognosis. Thus, this new molecular classification has deepened our understanding of the molecular characteristics of GCs and will benefit the targeted therapy.

### Therapeutic implications of the TCGA molecular classification

#### The CIN subtype

**Fig. 2:**
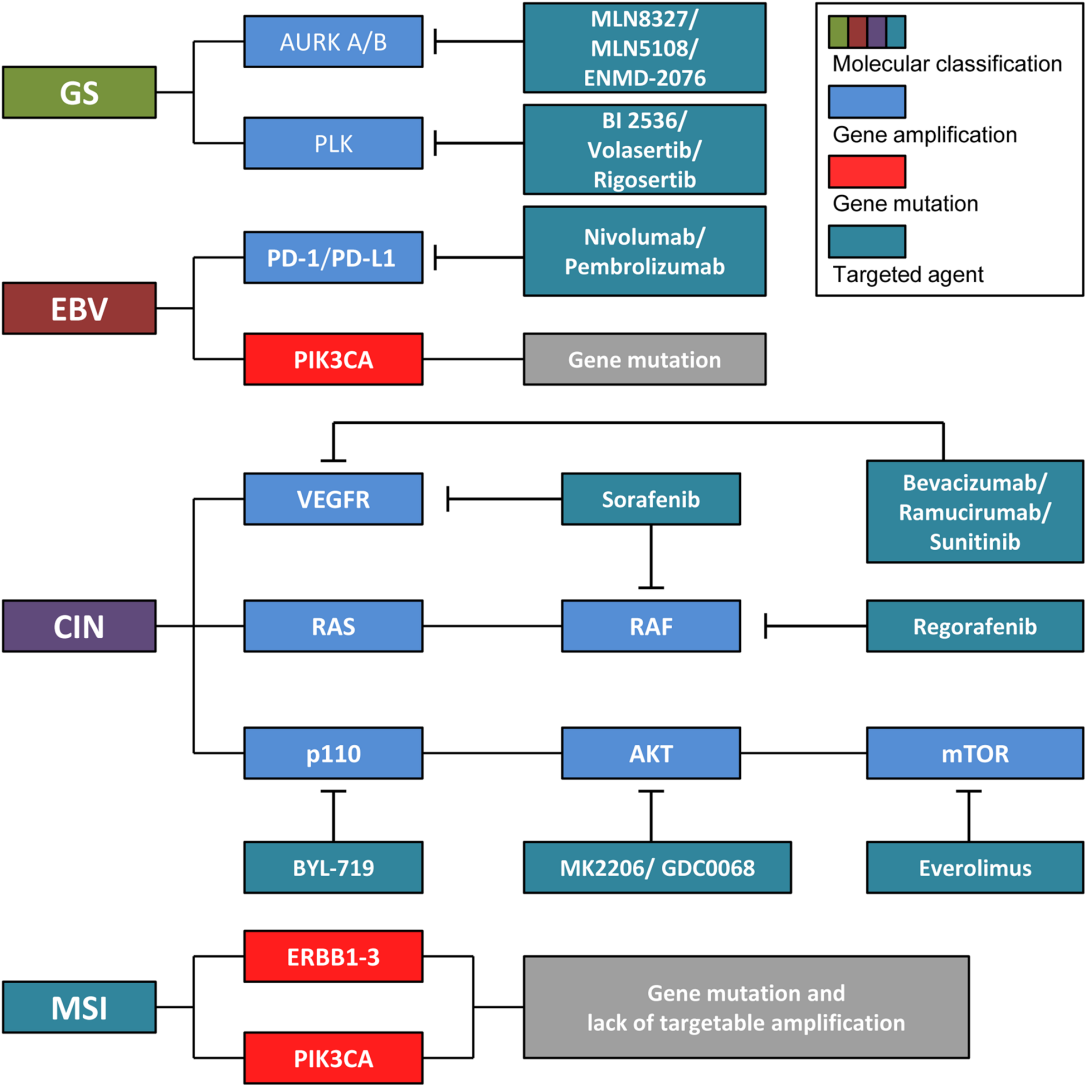
The implications of the TCGA molecular classification of GCs for individualized therapeutics. *AURK* aurora kinase, *PLK* polo-like kinase, *VEGFR* vascular endothelial growth factor receptor, *AKT* v-akt murine thymoma viral oncogene homolog 1, *mTOR* mechanistic target of rapamycin, *ERBB* erb-b2 receptor tyrosine kinase. Other abbreviations as in Fig. 1

**Fig. 3:**
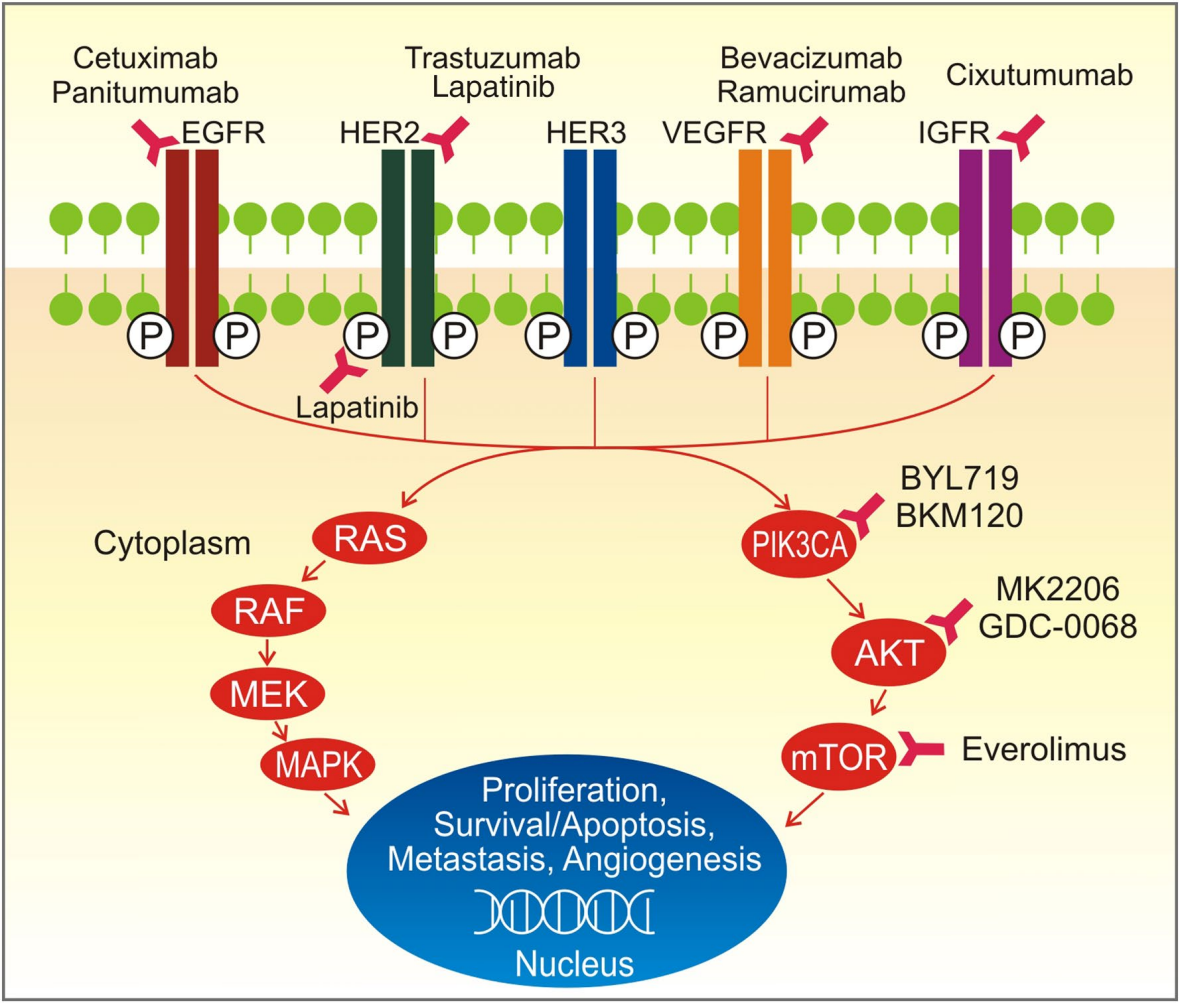
The current targeted therapies for advanced GCs. EGFR epidermal growth factor receptor, *HER2* erb-b2 receptor tyrosine kinase 2, *IGFR* insulin-like growth factor receptor, *MEK* MAP kinse-ERK kinase, *MAPK* mitogen-activated protein kinase. Other abbreviations as in Figs. 1, 2

The TCGA molecular classification provides a number of clinical impacts on individualized therapeutics (Fig. 2) [14]. In the CIN subtype, the TCGA network identified genomic amplifications of RTKs and resistance to audiogenic seizures (*RAS*), many of which are targetable. In the recent decade, the RTK pathway has been heavily investigated [32, 33] and is regarded as a promising candidate target for individualized therapy for GCs (Fig. 3). *EGFR* overexpression is associated with an aggressive phenotype and short survival [34]. However, similar to the results in patients with colorectal cancer, the expression levels of *EGFR* did not associate with treatment efficacy on GCs. *HER2* is overexpressed in various cancer types and acts as an oncogene involved in the regulation of cell proliferation, differentiation, motility, and apoptosis [33]. In breast cancer, the overexpression of *HER2* is a poor prognosis marker for patients who underwent chemotherapy and endocrine therapy but is a positive predictive marker for patients who underwent adjuvant treatment with trastuzumab, a fully humanized monoclonal antibody. In GCs, after the preliminary phase II study [35], Bang et al. [36] completed a phase III ToGA trial, and this milestone study established trastuzumab as the first biological therapy. In this trial that demonstrated survival benefits in GC patients, the median overall survival (OS) was 13.8 months in those allocated to trastuzumab plus chemotherapy compared with 11.1 months in those assigned to chemotherapy alone. In addition to the primary endpoint, the median progression-free survival (PFS, 6.7 vs. 5.5 months) and radiological response rate (47% vs. 35%) were also improved with trastuzumab therapy. The phase III TYTAN study compared paclitaxel alone or in combination with lapatinib in HER2-positive GCs in the second-line setting in Asian patients [37]. The median OS was 11 months with paclitaxel plus lapatinib compared with 8.9 months with paclitaxel alone. Selected completed phase III clinical trials about targeted therapies in advanced GCs are shown in Table 2 [36–41].

**Table 2:** Completed phase III clinical trials of targeted therapies for advanced GCs

Target	Clinical trial	Inhibitor	Combination treatment	No. of cases	Primary endpoint	Reference
HER2	ToGA	Trastuzumab	Capecitabine/5-fluorouracil + cisplatin	594	OS (13.8 months)	[36]
	TyTAN	Lapatinib	Paclitaxel	261	OS (11 months)	[37]
EGFR	EXPAND	Cetuximab	Capecitabine/capecitabine + cisplatin	904	PFS (4.4 months)	[38]
	REAL-3	Panitumumab	Best supportive care	463	PFS (2 months)	[39]
VEGFR	AVAGAST	Bevacizumab	Capecitabine + cisplatin	774	OS (12.1 months)	[40]
	REGARD	Ramucirumab	Best supportive care	355	PFS (5.2 months)	[41]

*HER2* erb-b2 receptor tyrosine kinase 2, *EGFR* epidermal growth factor receptor, *VEGFR* vascular endothelial growth factor receptor, *OS* overall survival, *PFS* progression-free survival

Angiogenesis may be highly relevant to the CIN subtype based on the recurrent amplification of vascular endothelial growth factor (*VEGF*) gene. Studies suggest that angiogenesis is a malignant hallmark, and angiogenesis has served as a common therapeutic target [42, 43]. The vascular endothelial growth factor receptor (VEGFR)-targeting antibody ramucirumab has demonstrated antitumor effects on GCs and was approved by the Food and Drug Administration (FDA) in the United States for advanced gastric or gastroesophageal junction adenocarcinoma patients with progression on fluoropyrimidine- or platinum-containing chemotherapy. REGARD is a phase III trial evaluating ramucirumab plus best supportive care versus placebo in patients with advanced GCs who have progressed after first-line chemotherapy [41]. In this trial, ramucirumab significantly prolonged the median OS compared with the placebo. In another AVAGAST phase III trial, plasma VEGFA was a strong biomarker candidate for predicting clinical outcome in patients with advanced GCs treated with bevacizumab [40].

#### The EBV subtype

The EBV subtype highlights the viral etiology of GCs; the TCGA characterization of this subtype suggests potential therapeutic targets for this subgroup of cancers. Of therapeutic importance, there is a strong predilection for *PIK3CA* mutation in EBVaGCs, with non-silent *PIK3CA* mutations noted in 80% of these cases. In contrast, tumors in other subtypes displayed fewer *PIK3CA* mutations (range from 3% to 42%). Preclinical work has demonstrated that hotspot *PIK3CA* mutations led to constitutive PIK3CA pathway signaling in the absence of growth factors [44–46]. Persistent PIK3CA signaling is a significant component of acquired resistance to upstream inhibitors [47, 48], including BYL-719 (anti-p110), MK2206 (anti-AKT), and GDC-0068 (anti-AKT). Thus, the TCGA molecular classification will be very important for future work to evaluate how the EBV-positive and -negative GCs respond to the available PIK3CA inhibitors. Unique treatment strategies for *PIK3CA*-mutated GCs must be explored.

TCGA analysis also showed that PD-1/PD-L1 was overexpressed in EBVaGCs. It has been reported that patients with cancers expressing high levels of PD-L1 were more sensitive to anti-PD-1 therapy than those with low levels of PD-L1 [49–51]. PD-1, a T cell co-inhibitory receptor, plays an important role in the process of cancer cell escape from the host’s immune system. The PD-L1/PD-1 axis can protect cancers from T-effector cells and help maintain an immunosuppressive microenvironment [52, 53]. The above results suggest that PD-L1 antagonists represent new therapeutic options for human cancers, especially advanced solid tumors. The FDA recently approved two anti-PD-1 monoclonal antibodies, Opdivo (also known as nivolumab) and Keytruda (also known as pembrolizumab), to treat human cancers. In addition, several monoclonal antibodies to either PD-1 or PD-L1 are undergoing development in numerous clinical trials. Nivolumab was the first monoclonal antibody targeting PD-1 to exhibit significant clinical activity in solid tumors [54, 55]. Nivolumab has a consistent objective response rate (ORR) and also extended OS in several clinical studies in patients with melanoma [56, 57] or non-small cell lung carcinoma (NSCLC) [58]. Nivolumab was approved by the FDA to treat both advanced melanoma and NSCLC. Pembrolizumab has demonstrated efficacy and safety similar to nivolumab in advanced melanoma [59, 60]. More recently, pembrolizumab also demonstrated efficacy in patients with advanced NSCLC [61] and has shown promising effects on other solid tumors, including GC [53]. Muro et al. [53] reported their preliminary results from the KEYNOTE-012 trial (NCT01848834) at the American Society of Clinical Oncology (ASCO) conference. They assessed the safety and efficacy of the anti-PD-1 monoclonal antibody pembrolizumab in patients with advanced GCs. In this study, the median time-to-response was 8 weeks (range, 7–16 weeks), with a median response duration of 24 weeks. PD-L1 expression levels were associated with the ORR. The 6-month PFS rate was 24%, and the 6-month OS rate was 69%. It was concluded that pembrolizumab demonstrated manageable toxicity and promising antitumor activity in advanced GCs [53]. These results support the ongoing development of anti-PD-1 therapy for GCs.

#### The GS and MSI subtypes

The GS subtype exhibited elevated expression of cell adhesion pathways, including the B1/B3 integrins, syndecan-1-mediated signaling, and angiogenesis-related pathways [14]. These results suggest additional candidate therapeutic targets, including Aurora kinase (*AURK*)*A/B* and polo-like kinase (*PLK*). In contrast, MSI cases generally lacked targetable amplifications, and mutations in *ERBB1*-*3* and *PIK3CA* were noted, with many mutations at “hotspot” sites observed in other types of cancers [14]. AURKA and AURKB are two main members of the aurora kinase family. AURKA plays an important role during cell mitosis and is frequently amplified in several tumors, including GC [62], colorectal cancer [63], pancreatic cancer [64], esophageal cancer [65], and lung cancer [66]. MLN8237, also known as alisertib, is a second-generation derivative of the initial small molecule MLN8054. Both MLN8237 and MLN8054 act as highly specific adenosine triphosphate (ATP)-competitive AURKA inhibitors. In addition, MLN8237 can target AURKB at high doses [67, 68]. It is an effective drug with high specificity that works in various models and exhibits limited off-target activity. There are more than 40 clinical trials with MLN8237 in several cancer types [69]. In some of the clinical trials, MLN8237 has been tested in combination with other drugs, such as cetuximab (NCT01540682, phase I) and docetaxel (NCT01094288, phase I). In advanced GCs, a recent phase II clinical trial of MLN8237 revealed that 9% of patients responded to this therapy [69]. Other AURKA/B inhibitors, such as MK-5018 and ENMD-2076, are currently being evaluated in phase I and II clinical trials as a single agent or in combination with other therapeutic agents [69].

PLKs, mitotic kinases of the polo family, play a critical role in the normal cell cycle, and their overexpression is involved in the pathogenesis of multiple human cancers [70–74]. Among PLKs, PLK1 is overexpressed in approximately 80% of human tumors, including GC, and is associated with a poor prognosis [70, 75, 76]. Currently, inhibitors of PLK1 represent a new class of cytotoxic agents. BI 2536 is a highly specific and potent small-molecule PLK1 inhibitor. In an open-labelled phase I study by Hofheinz et al. [77], BI 2536 administered in the treatment schedule demonstrated adequate safety in patients with advanced GCs. Volasertib (BI 6727) is a potent and selective PLK inhibitor that induces mitotic arrest and apoptosis. In phase I trials of both Asian and Caucasian patients with advanced solid cancers, including GC, volasertib demonstrated anti-cancer activity with a generally manageable safety profile [78, 79]. Phase II monotherapies and combination trials of volasertib are currently ongoing.

## Conclusions

The deep understanding of GC molecular characterizations has led to new therapeutic strategies. Furthermore, the TCGA molecular classification that shows GC heterogeneity and distinct salient genomic features provides a guide to targeted agents for GC individualized therapy. Specifically for immune checkpoints, PD-L1/PD-1 appears to be Achilles’ heels for the EBV subtype of GC. However, it should also be noted that much work is needed to fully understand the clinical impact of this new classification. Therefore, a major unfulfilled task is to determine the clinical associations of the molecular signatures given that the cases in the TCGA cohort lacked sufficient clinical follow-up data. Nevertheless, the TCGA report is expected to provide valuable foundation and motivation to explore and refine molecular classification and tailored therapies to significantly decrease mortality and prolong survival of GC patients.
